# ERG and c-MYC regulate a critical gene network in BCR::ABL1-driven B cell acute lymphoblastic leukemia

**DOI:** 10.1126/sciadv.adj8803

**Published:** 2024-03-08

**Authors:** Kira Behrens, Natalie Brajanovski, Zhen Xu, Elizabeth M. Viney, Ladina DiRago, Soroor Hediyeh-Zadeh, Melissa J. Davis, Richard B. Pearson, Elaine Sanij, Warren S. Alexander, Ashley P. Ng

**Affiliations:** ^1^ Blood Cells and Blood Cancer Division, The Walter and Eliza Hall Institute of Medical Research, Parkville, Australia.; ^2^ Department of Medical Biology, University of Melbourne, Parkville, Australia.; ^3^ Division of Cancer Research, Peter MacCallum Cancer Centre, Melbourne, Australia.; ^4^ Immunology Division, The Walter and Eliza Hall Institute of Medical Research, Parkville, Australia.; ^5^ Bioinformatics Division, Walter and Eliza Hall Institute of Medical Research, Parkville, Australia.; ^6^ Department of Clinical Pathology, University of Melbourne, Parkville, Australia.; ^7^ The Diamantina Institute, The University of Queensland, Woolloongabba, Australia.; ^8^ The South Australian Immunogenomics Cancer Institute, The University of Adelaide, Adelaide, Australia.; ^9^ Sir Peter MacCallum Department of Oncology, University of Melbourne, Parkville, Australia.; ^10^ Department of Biochemistry and Molecular Biology, Monash University, Clayton, Australia.; ^11^ Department of Biochemistry and Molecular Biology, University of Melbourne, Parkville, Australia.; ^12^ St. Vincent’s Institute of Medical Research, Fitzroy, Australia.; ^13^ Department of Medicine, St. Vincent’s Hospital, University of Melbourne, Parkville, Australia.

## Abstract

Philadelphia chromosome–positive B cell acute lymphoblastic leukemia (B-ALL), characterized by the *BCR::ABL1* fusion gene, remains a poor prognosis cancer needing new therapeutic approaches. Transcriptomic profiling identified up-regulation of oncogenic transcription factors ERG and c-MYC in *BCR::ABL1* B-ALL with ERG and c-MYC required for *BCR::ABL1* B-ALL in murine and human models. Profiling of ERG- and c-MYC–dependent gene expression and analysis of ChIP-seq data established ERG and c-MYC coordinate a regulatory network in *BCR::ABL1* B-ALL that controls expression of genes involved in several biological processes. Prominent was control of ribosome biogenesis, including expression of RNA polymerase I (POL I) subunits, the importance of which was validated by inhibition of *BCR::ABL1* cells by POL I inhibitors, including CX-5461, that prevents promoter recruitment and transcription initiation by POL I. Our results reveal an essential ERG- and c-MYC–dependent transcriptional network involved in regulation of metabolic and ribosome biogenesis pathways in *BCR::ABL1* B-ALL, from which previously unidentified vulnerabilities and therapeutic targets may emerge.

## Teaser

ERG and c-MYC regulate metabolic and ribosome biogenesis pathways in pre–B-ALL, revealing potential therapeutic targets.

## INTRODUCTION

Acute lymphoblastic leukemia (ALL) is a highly aggressive cancer. Numerous genetic subtypes of ALL have been identified, including Philadelphia chromosome–positive B cell ALL (Ph^+^B-ALL) that comprises ~25 to 30% of all adult ALL. Ph^+^B-ALL is characterized by the *t*(9;22) chromosomal translocation that generates the *BCR::ABL1* fusion gene resulting in abnormal tyrosine kinase signaling in B lymphoid progenitors that drives leukemia development. Ph^+^B-ALL remains a poor-prognosis cancer (*1*–*3*) despite the improved outcomes achieved by targeting the driver BCR::ABL1 fusion protein with tyrosine kinase inhibitors (TKIs) (*4*). More than 50 to 60% of patients relapse (*5*–*7*), including those receiving newer immunotherapeutic approaches. A better understanding of disease pathogenesis would assist rational development of new targeted therapeutic approaches.

While additional genomic lesions in *BCR::ABL1* B-ALL have been identified, these are largely loss-of-function alleles, including the B cell transcription factor genes *IKZF1* (*IKAROS*), *PAX5*, and *EBF1*, that are clinically associated with poorer disease outcomes (*8*–*11*). Loss of these factors in preclinical models has been associated with accelerated leukemia expansion that is proposed to occur via loss of their metabolic gatekeeper function (*12*), suggesting that these genes and members of their gene regulatory networks are unlikely to represent viable molecular candidates as direct therapeutic targeting of loss-of-function alterations is problematic.

We sought to specifically identify gain of function and thus potentially targetable alterations that contribute to Ph^+^B-ALL pathogenesis, including those that may not be identified through diagnostic genomic assays. To do this, we used a murine model of *BCR::ABL1* B-ALL to identify transcription factors and gene regulatory networks required for leukemia initiation and progression. Transcriptional profiling revealed up-regulation of *ERG* and *c-MYC* in murine and human *BCR::ABL1* B-ALL, and, in murine and human models, ERG and c-MYC were required for *BCR::ABL1* B-ALL. Profiling of ERG- and c-MYC–dependent gene expression and analysis of chromatin immunoprecipitation sequencing (ChIP-seq) data established that ERG and c-MYC coordinate a regulatory network in *BCR::ABL1* B-ALL directly controlling expression of genes enriched for involvement in several cellular processes. Prominent was control of ribosome biogenesis, including direct regulation of RNA polymerase I (POL I) subunits, the role of which was validated by inhibition of *BCR::ABL1* cells and other genomic subtypes by POL I inhibitors.

## RESULTS

### *ERG, c-MYC*, and downstream gene targets are highly expressed in Ph^+^B-ALL

**Fig. 1.**
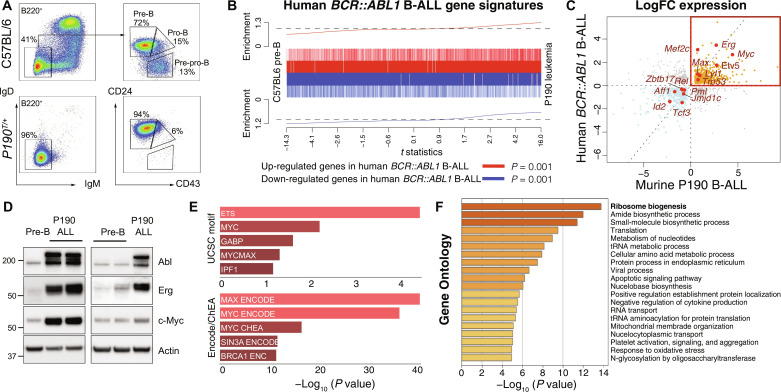
Gene expression analysis in *BCR::ABL1* B-ALL. (**A**) Representative flow cytometry plots of bone marrow B-lymphoid populations from C57BL/6 wild-type (WT) control mice and leukemic P190 mice with the percentage of cells in each gated population indicated. The immunoglobulin D (IgD)/IgM profile is from B220^+^ gated cells; the CD24/CD43 profile from B220^+^ IgD^−^ IgM^−^ gated cells. (**B**) Barcode enrichment plots of up-regulated (red) and down-regulated (blue) genes in human Ph^+^B-ALL (GSE5314, *n* = 37) compared to murine P190 B-ALL gene expression changes (*n* = 5). *P* values were calculated by ROAST gene set testing. (**C**) Scatter plot of log_2_ fold change (LogFC) in expression of orthologous genes in human Ph^+^B-ALL (GSE5314) and murine P190 B-ALL compared to normal pre-B cells. Transcription factors from Kyoto Encyclopedia of Genes and Genomes transcriptional deregulation in cancer (ko05202) and the quadrant of up-regulated genes in both human and murine *BCR::ABL1* B-ALL are highlighted in red. (**D**) Western blot analysis of Abl, Erg, and c-Myc in pre-B cells, compared to three independent *P190^T/+^*cell lines. Actin serves as loading control. (**E**) In silico motif discovery of up-regulated genes in human and murine *BCR::ABL1* B-ALL [Enrichr, https://maayanlab.cloud/Enrichr/; with UCSC transcription factor ENCODE motif discovery (*72*) and ENCODE Consensus Target Genes and ChEA Transcription Factor ChIP-X (*73*)]. (**F**) Gene Ontology (GO) analysis of up-regulated genes in human and murine *BCR::ABL1* B-ALL (Metascape, https://metascape.org; see also table S1).

We initially determined the transcriptional changes associated with leukemic transformation in *BCR::ABL1* B-ALL in the P190 transgenic mouse model that expresses a human *BCR::ABL1* transgene under the control of the metallothionein promoter (*13*). This transgenic mouse develops a highly penetrant acute pre-B cell (precursor B cell) lymphoblastic leukemia that closely phenocopies human disease. The murine model is characterized by expansion of pre-B cells in the bone marrow, typically between 5 and 8 weeks of age, and a B-lineage differentiation block resulting in a deficiency of maturing immunoglobulin M (IgM)^+^IgD^+^ B cells (Fig. 1A).

Pre-B cells from leukemia-bearing P190 mice were isolated, and genome-wide gene expression profiling was undertaken by bulk RNA sequencing (RNA-seq) comparing leukemic cells to control (non-transformed) C57BL/6 pre-B cells. This analysis identified significant transcriptional deregulation of 9258 genes in P190 pre-B leukemic cells (4720 up and 4538 down; table S1). This included down-regulation of genes implicated in B-lineage differentiation, such as *Ikzf1* (*14*, *15*), *Ikzf3* (Aiolos) (*16*), *Pax5* (*17*, *18*), *Tcf3*(E2A) (*19*, *20*), and *Irf4* and *Irf8* (*21*) (fig. S1A) that may contribute to the differentiation block observed in P190 leukemic mice. Of note, loss-of-function alleles of several of these down-regulated genes have been identified in human ALL validating the use of this model (*8*, *9*).

Comparison of transcriptional changes in murine P190 leukemic pre-B cells to publicly available gene expression data from human *BCR::ABL1* B-ALL (GSE5314) (*22*) revealed significant correlation with transcriptional changes in human B-ALL (Fig. 1, B and C).

Examination of transcription factors from the Kyoto Encyclopedia of Genes and Genomes deregulated in cancer gene set (ko05202) identified up-regulation of several oncogenic transcription factors involved in B cell differentiation, including the E26 Transformation-Specific (ETS) family transcription factor *ERG* (*23*), the β helix–loop–helix transcription factor c-*MYC* (*24*) and its cooperative transcriptional partner *MAX* (*25*) in both murine and human *BCR::ABL1* B-ALL (Fig. 1C). Increased protein levels of ERG and MYC were observed in P190 B-ALL cell lines compared to control pre-B cells (Fig. 1D).

Consistent with regulation by ERG and c-MYC of gene networks required for *BCR::ABL1* B-ALL leukemogenesis, significant enrichment for the ETS, c-MYC, and MAX binding motifs was found in genes up-regulated in murine and human *BCR::ABL1* B-ALL (Fig. 1E). Gene Ontology (GO) analysis of up-regulated genes in murine and human *BCR::ABL1* B-ALL showed that the most significantly enriched gene sets were associated with ribosome biogenesis, as well as processes such as amide biosynthesis and nucleotide, tRNA, and amino acid metabolism (Fig. 1F and table S1). Up-regulation of ribosome biogenesis in *BCR::ABL1* leukemia was confirmed by significantly increased transcription of the POL I–dependent 47*S*/45*S* precursor ribosomal RNA (rRNA) internal transcribed spacer in P190 leukemia cells compared to wild-type pre-B cells (fig. S1B).

### ERG and c-MYC are critical for Ph^+^B-ALL development

To explore the functional significance of ERG and c-MYC in Ph^+^B-ALL, we first examined whether co-occurrence of *BCR::ABL1* and *ABL1* class fusions occurred with genomic variants of *ERG* and *c-MYC* in a cohort of human ALL (St. Jude, PeCan, accessed 21 January 2022) (*26*). While loss-of-function variants and copy number loss of *ERG* and, to a smaller extent, *c-MYC* were identified in this cohort of B-ALL, these did not co-occur with *BCR::ABL1* or *ABL1* class fusions (fig. S1C). Examination of CRISPR-Cas9–based gene dependency screening by the Cancer Dependency Map initiative (*27*) demonstrated that, while gene dependency for *ERG* and c-*MYC* could be variably identified for several genomic subtypes in B-ALL cell lines, no *BCR::ABL1* class cell lines had been assessed (fig. S1D).

**Fig. 2.**
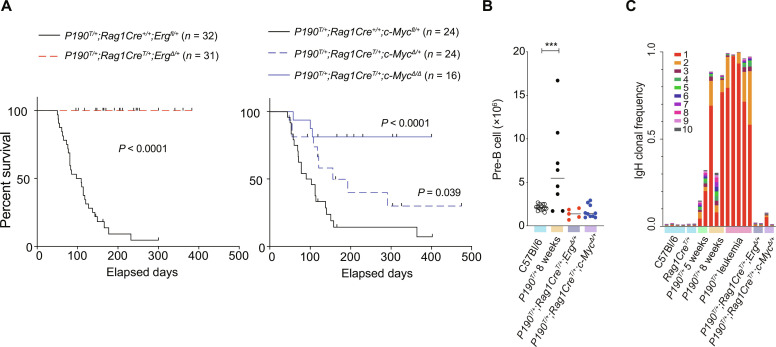
Reduction of ERG or c-MYC impairs initiation of *BCR::ABL1* B-ALL. (**A**) Kaplan-Meier curves showing time to ethical endpoint due to leukemia in P190 transgenic mice lacking one allele of *Erg* (*P190^T/+^;Rag1Cre^T/+^;Erg*^Δ*/+*^, left; *n* = 31) and one (*P190^T/+^;Rag1Cre^T/+^;Myc^Δ/+^*; *n* = 24) or both (*P190^T/+^;Rag1Cre^T/+^;Myc^Δ/Δ^*; *n* = 16) alleles of *c-Myc* (right) in lymphoid cells. *P* values were calculated by log-rank test*.* (**B**) Number of pre-B cells per femur at 8 weeks of age in *P190^T/+^;Rag1Cre^T/+^;Erg^Δ/+^* (*n* = 5) and *P190^T/+^;Rag1Cre^T/+^;Myc^Δ/+^* (*n* = 10) mice compared to P190 (*n* = 8) and control C57BL/6 (*n*= 14) mice. ***P ≤ 0.05 by Dunnett’s multiple comparisons corrected one-way analysis of variance (ANOVA). (**C**) Immunoglobulin heavy-chain gene clonotyping analysis of pre-B cells from C57BL/6 WT controls (*n* = 4 replicates) and *P190^T/+^* mice at 5 (*n* = 2 replicates) and 8 (*n* = 3 replicates) weeks of age, *P190^T/+^* leukemia cells from symptomatic mice (*n* = 5 replicates), and *P190^T/+^;Rag1Cre^T/+^;Erg^Δ/+^* (*n* = 2 replicates) and *P190^T/+^;Rag1Cre^T/+^;Myc^Δ/+^* (*n* = 2 replicates) pre-B cells, demonstrating relative frequency of 10 most frequent clones [MiXCR version 3.0.6, accessed 21 January 2021; (*63*)].

To directly address the requirement for ERG and c-MYC in *BCR::ABL1* B-ALL, we generated P190 mice in which the *Erg* or *c-Myc* genes were deleted from the Common lymphoid progenitor (CLP) stage of lymphopoiesis using a *Rag1-Cre* conditional knockout approach (*28*). Deletion of a single allele of *Erg* (*P190^T/+^;Rag1Cre^T/+^;Erg*^∆*/+*^) was sufficient to prevent the development of P190 B-ALL in this model. Deletion of one *c-Myc* allele (*P190^T/+^;Rag1Cre^T/+^;c-Myc*^Δ*/+*^) also significantly delayed leukemia development, and this delay was more pronounced in the absence of both *c-Myc* alleles (*P190^T/+^;Rag1Cre^T/+^;c-Myc*^Δ*/*Δ^) (Fig. 2A). At 5 weeks of age, pre-B cell numbers in P190 bone marrow were similar to wild-type mice, whereas, at 8 weeks of age, before overt symptoms of P190 disease, a significant proportion of P190 mice had developed an abnormal accumulation of pre-B cells (fig. S1E). In contrast, in P190 mice lacking one copy of *Erg* or *c-Myc*, no expansion of pre-B cells was seen, with pre-B cell numbers in 8-week-old *P190^T/+^;Rag1Cre^T/+^;Erg*^Δ*/+*^ and *P190^T/+^;Rag1Cre^T/+^;c-Myc^Δ/+^* mice comparable to those seen in C57BL/6 controls (Fig. 2B). To determine the impact of loss of *Erg* or *c-Myc* alleles on clonal expansion during *BCR::ABL1* leukemogenesis, immunoglobulin heavy-chain (*Igh*) gene clonotyping analysis was performed on bulk RNA-seq data obtained from primary pre-B cells. In P190 mice, leukaemogenesis was associated with dominant clones arising at 5 and 8 weeks of age, with one clone often becoming dominant in mice developing overt leukemia (Fig. 2C). In contrast, quantitative analysis of the 10 most frequent *Igh* clones revealed no significant dominant clonal expansion in *P190 ^T/+^;Rag1Cre^T/+^;Erg*^Δ*/+*^ mice at 8 weeks of age. Similarly, dominant clonal expansion was not observed in *P190^T/+^;Rag1Cre^T/+^;c-Myc^Δ/+^* mice. Together, these data demonstrate high expression of *ERG* and *c-MYC* in *BCR::ABL1* B-ALL compared to non-transformed pre-B cells and that these transcription factors are necessary for pre-B cell clonal expansion and subsequent leukemia development.

### ERG and c-MYC contribute to *BCR::ABL1* B-ALL maintenance

**Fig. 3.**
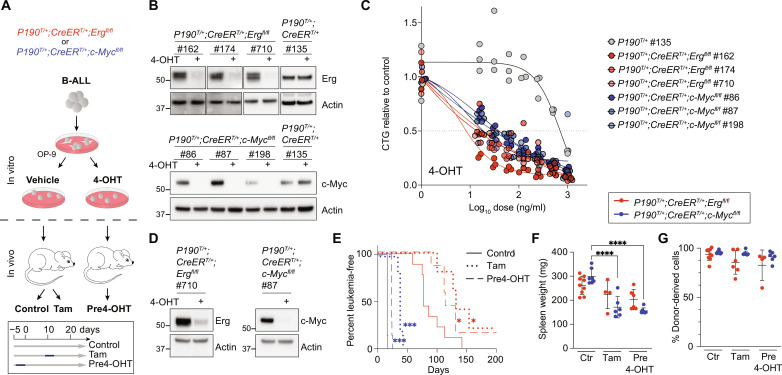
The role of ERG and c-MYC in established murine *BCR::ABL1* B-ALL. (**A**) B-ALL cell lines were derived from mice of indicated genotypes. For in vitro studies, cell lines were cultured in 4-OHT or vehicle. For transplantation studies, cell lines were cultured in 4-OHT (pre4-OHT) or vehicle for 5 days before transplantation of 1 × 10^6^ cells into sublethally irradiated recipient mice. Recipients injected with vehicle-treated cells were divided into a control cohort (untreated) and a cohort receiving tamoxifen (TAM) on days 8 to 11 (Tam). (**B**) Western blot analysis of Erg or c-Myc in three independent *P190^T/+^;CreER^T/+^;Erg^fl/fl^* or *P190^T/+^;CreER^T/+^;c-Myc^fl/fl^* cell lines cultured ± 4-OHT (250 ng/ml) for 96 hours. Actin loading control. (**C**) Cell titer glow assay of three independent *P190^T/+^;CreER^T/+^;Erg^fl/fl^* (red) and *P190^T/+^;CreER^T/+^;c-Myc^fl/fl^* (blue) cell lines compared to *P190^T/+^;CreER^T/+^* (gray) control lines treated with the indicated concentrations of 4-OHT. Shown are the means of three independent experiments performed in triplicates. (**D**) Western blot analysis of Erg (left) or c-Myc (right) in samples of 4-OHT–pretreated or vehicle-treated *P190^T/+^;CreER^T/+^;Erg^fl/fl^* or *P190^T/+^;CreER^T/+^;c-Myc^fl/fl^* cells analyzed immediately before transplantation (day 0). Actin serves as loading control. (**E**) Kaplan-Meier curves of mice transplanted with untreated cells [solid line, *n* = 9 (red) and *n* = 7 (blue)], 4-OHT–pretreated cells [dashed line, *n* = 7 (red) and *n* = 7 (blue)] and in vivo TAM-treated mice [dotted line, *n* = 5 (red) and *n* = 6 (blue)]. Both *P190 ^T/+^;CreER^T/+^;Erg^fl/fl^* (red) and *P190^T/+^;CreER ^T/+;^c-Myc ^fl/fl^* (blue) cells were assessed. Data from two independent experiments. *P* values were calculated by Gehan-Breslow-Wilcoxon test. *P ≤ 0.05; ***P ≤ 0.001. (**F**) Spleen weights and (**G**) percentage donor-derived bone marrow cells as measured by CD45.2 expression from leukemic mice [see (E)]. Each dot represents a mouse (*n* = 4 to 9). Error bars represent SD. *P* values were calculated by Dunnett’s multiple comparisons corrected one-way ANOVA. ****P ≤ 0.0001. Ctr, control.

We next assessed the role of ERG and c-MYC in sustaining established *BCR::ABL1* leukemia. We derived multiple independent cell lines from leukemic P190 mice carrying either floxed *Erg* (*Erg^fl/fl^*) or *c-Myc* (*c-Myc^fl/fl^*) alleles in addition to the *CreERT2* transgene (*29*) to allow 4-hydroxy-tamoxifen (4-OHT)–dependent deletion of the floxed alleles (Fig. 3A, top). Upon 4-OHT treatment, reduced expression of ERG or c*-*MYC was observed in the respective cell lines, in which *Erg* or *c-Myc* was conditionally deleted (Fig. 3B). Dose-responsive inhibition of these ERG and c-MYC–deficient leukemic cell lines was observed, an effect not seen in in *P190^T/+^;CreER^T/+^* control leukemia cells (Fig. 3C).

We next investigated the requirement for ERG and c-MYC in *BCR::ABL1* leukemia maintenance in vivo. Individual *P190^T/+^;CreER*^T/+^;*Erg^fl/fl^* and *P190^T/+^;CreER^T/+^;c-Myc^fl/fl^* leukemia cell lines were pretreated with either 4-OHT (pre4-OHT) resulting in significant loss of *Erg* or *c-Myc* expression (Fig. 3D) or vehicle control and then transplanted into irradiation-conditioned recipients. Mice that received vehicle-treated cells were then either given tamoxifen (TAM) at day 8 or left untreated (control group) (Fig. 3A, bottom). In recipients of cells that are ERG- or MYC-deficient, by either 4-OHT pretreatment or in vivo TAM administration, the time to ethical endpoint due to leukemia was prolonged relative to control mice (Fig. 3E). Splenomegaly was observed to be significantly reduced in cohorts that received c-MYC–deficient cells (Fig. 3F), while the proportion of donor cells in bone marrow was observed to be consistently high in all groups (Fig. 3G). Notably, leukemia that developed in mice transplanted with *P190^T/+^;CreER*^T/+^;*Erg^fl/fl^* and *P190^T/+^;CreER^T/+^;c-Myc^fl/fl^* cell lines following 4-OHT treatment or TAM treatment in vivo demonstrated incomplete and variable reduction in ERG or c-MYC expression in the diseased bone marrow cells (fig. S2A), suggesting in vivo expansion of leukemia cells that had escaped efficient Cre-mediated gene recombination had occurred.

**Fig. 4.**
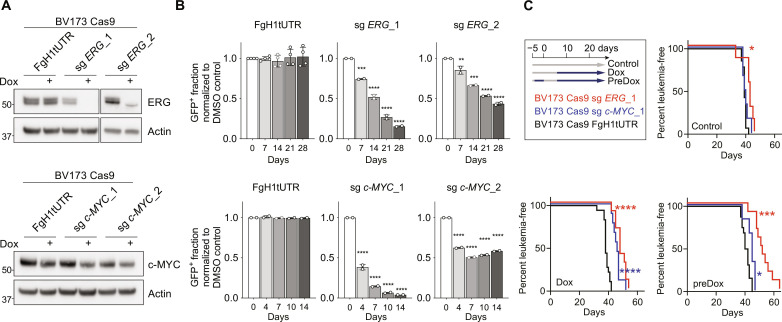
The role of ERG and c-MYC in human *BCR::ABL1* B-ALL. (**A**) Western blot analysis of ERG or c-MYC in BV173 cells transduced to express Cas9 and one of two independent Dox-inducible guide RNAs targeting *ERG* (sg ERG_1 and sg ERG_2) or c-*MYC* (sg c-MYC_1 and sg c-MYC_2), respectively. Actin serves as loading control. Cells were treated with Dox (+) (100 ng/ml) or vehicle for 96 hours as indicated. Cells containing the empty FgH1UTR vector served as controls. (**B**) In vitro proliferation of Dox-treated BV173 Cas9 sg ERG, BV173 Cas9 sg MYC, or BV173 Cas9 FgH1UTR cells relative to dimethyl sulfoxide (DMSO)–treated control cells. Shown are the means of two independent experiments. Error bars represent SD. *P* values were calculated by one-way ANOVA with Dunnett’s multiple comparisons test. **P ≤ 0.01; ***P ≤ 0.001; ****P ≤ 0.0001. (**C**) BV173 Cas9 guide RNA co-expressing cells were cultured with (preDox) or without Dox (100 ng/ml) for 5 days. Cells (1 × 10^6^) were injected per NOD.Cg-*Prkdc^scid^IL2rg^tm1Wjl^*/Szj (NSG) mouse on day 0. Seven days after transplantation, recipients of pretreated cells (preDox) and half of the cohort transplanted with vehicle-treated BV173 cells were fed Dox-impregnated pellets (Dox), and the other half were left untreated (control). Kaplan-Meier curves show time to ethical endpoint due to leukemia in mice transplanted with BV173 Cas9 sg ERG (red), BV173 Cas9 sg MYC (blue), or BV173 Cas9 FgH1UTR (black) cells [control: *n* = 7 (red), *n* = 6 (blue), and *n* = 14 (black); Dox: *n* = 10 (red), *n* = 9 (blue), and *n* = 19 (black); preDox: *n* = 10 (red), *n* = 6 (blue), and *n* = 15 (black)] from three independent experiments. *P* values were calculated by Gehan-Breslow-Wilcoxon test. *P ≤ 0.05; ***P ≤ 0.001; ****P ≤ 0.0001.

To confirm and extend our observations to human *BCR::ABL1* B-ALL cells, guide RNAs directed against human *ERG* or c-*MYC* were expressed via a doxycycline (Dox)–inducible lentiviral vector co-expressing green fluorescent protein, resulting in reduced ERG or c-MYC protein levels in respective Cas9-expressing human BV173 cells (Fig. 4A). Reduction of ERG or c-MYC expression by two independent guide RNAs for each gene resulted in a distinct competitive proliferative disadvantage in BV173 cells compared to empty vector controls (Fig. 4B). Similar results were obtained in a second *BCR::ABL1* B-ALL human cell line, SupB15 (fig. S2, B and C). Last, reduction of ERG or c-MYC either before transplantation (preDox) or treatment with Dox in vivo resulted in delayed BV173 tumor growth in transplanted mice compared to untreated controls or mice transplanted with empty vector expressing BV173 cells (control) (Fig. 4C).

In addition to *BCR::ABL1* B-ALL, prominent ERG and c-MYC expression is also found in other B-ALL subtypes (fig. S3A) and pre-B-ALL human cell lines, including Nalm6 and RS4:11 (fig. S3B), where binding of ERG and c-MYC to the *POLR1B* promoter can be observed (fig. S3C). As observed in *BCR::ABL1* B-ALL cell lines, genetic reduction of *ERG* or *c-MYC* expression in Nalm6 and RS4:11 cells resulted in a competitive proliferative disadvantage compared to empty vector controls (fig. S3, D to G).

### Identification of a gene network regulated by ERG and c-MYC in *BCR::ABL1* B-ALL

**Fig. 5.**
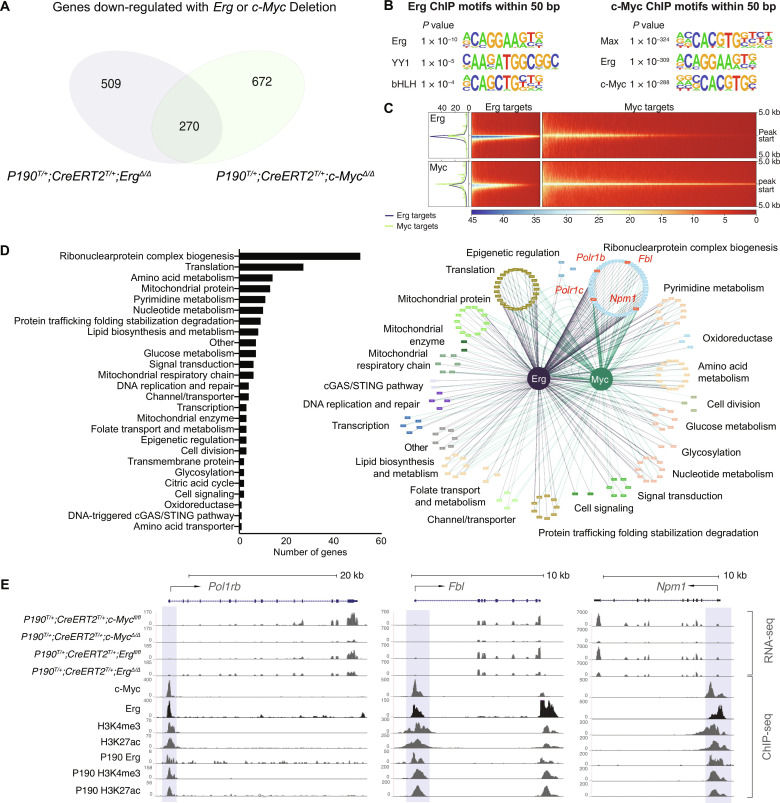
An Erg and c-Myc co-regulated transcriptional network in *BCR::ABL1* B-ALL. (**A**) Venn diagram of transcriptional changes due to deletion of *Erg* (*P190^T/+^;CreER^T/+^;Erg*^Δ*/Δ*^ versus *P190^T/+^;CreER^T/+^;Erg^fl/fl^*) or *c-Myc* (*P190^T/+^;CreER^T/+^;c-Myc^Δ/Δ^* versus *P190^T/+^;CreER^T/+^;c-Myc^fl/fl^*) in two independent cell lines per genotype, showing overlap of genes regulated by Erg and/or c-Myc (see also table S2). (**B**) Hypergeometric Optimization of Motif EnRichment (HOMER) transcription factor motif analysis of genomic regions bound by ERG (GSM3895108) and c-MYC (GSM1234475) from ChIP-seq analyses in pro-B cells. (**C**) Comparison of ERG and c-MYC binding centred to annotated transcriptional start sites (TSSs), showing log_2_ ratio of genome-wide Erg or c-Myc binding by ChIP-seq. (**D**) GO analysis of genes bound and down-regulated with both ERG and c-MYC deletion in *BCR::ABL1* B-ALL, showing the number of genes in each curated set (bar chart, left) and the Erg and c-Myc gene regulatory network (network diagram, right; see also table S3) with *Polr1b*, *Polr1c*, *Fbl*, and *Npm1* nucleolar genes highlighted. (**E**) RNA-seq and ChIP-seq tracks of *Polr1b*, *Fbl* and *Npm1* gene loci showing transcriptional changes associated with Erg (*P190^T/+^;CreER^T/+^;Erg^Δ/Δ^*) and c-Myc (*P190^T/+^;CreER^T/+^;Myc^Δ/Δ^*) deletion compared to *P190^T/+^;CreER^T/+^;Erg^fl/fl^* and *P190^T/+^;CreER^T/+^;Myc^fl/fl^* controls, and Erg and c-Myc binding to promoter regions (highlighted in blue) defined by H3K4me3 (GSM2255547) and H3K27Ac marks (GSM2255552), with tracks for independent ChIP-seq undertaken in the P190 *BCR::ABL1* cell line for ERG, H3K4me3, and H3K27Ac shown.

To define the gene networks regulated by ERG and c-MYC that facilitate leukaemogenesis, we first examined the gene expression changes upon deletion of either *Erg* or *c-Myc* in established *P190^T/+^;CreERT2^T/+^;Erg^fl/fl^* and *P190^T/+^;CreERT2^T/+^;c-Myc^fl/fl^* cell lines (table S2). Notably, there was overlap of differentially expressed genes upon *Erg* or *c-Myc* deletion in genetically independent cell lines. Of particular interest were genes down-regulated with both *Erg* or *c-Myc* deletion (Fig. 5A), as these genes may form part of a transcriptional gene network regulated by ERG and c-MYC, mediating the functional roles for these transcription factors in *BCR::ABL1* B-ALL during leukaemogenesis and in leukemia maintenance*.*

To define genes directly bound and transcriptionally regulated by ERG and c-MYC, publicly available pro-B cell ChIP-seq datasets for ERG (GSM3895108) and c-MYC (GSM1234475) were examined. An initial genome-wide motif analysis of the ERG ChIP-seq dataset identified enriched representation of the ERG motif at ERG binding sites as expected (Fig. 5B). In addition, enrichment of the β helix–loop–helix binding motif recognized by c-MYC (*30*) and the YY1 cohesin motif were also identified within 50 base pairs (bp) of ERG binding sites. For the complimentary analysis for c-MYC binding, the most highly enriched motifs within 50 bp of c-MYC–bound loci were not only motifs for the c-MYC binding partner MAX (*31*) as well as c-MYC, as expected, but also the ERG motif. We next focused on analysis of genome wide ERG and c-MYC binding to regions within 5 kb of a transcriptional start site (TSS). This analysis demonstrated overlap of ERG and c-MYC binding to a subset of genomic loci (Fig. 5C) and suggested that c-MYC and ERG co-localization at specific genomic loci may result in co-regulation of specific genes as part of a transcriptional network. Notably, no direct interaction between ERG and c-MYC was identified by co-immunoprecipitation (fig. S4A).

To explore this hypothesis further in *BCR::ABL1* B-ALL, differentially expressed genes that were found to be down-regulated upon both *Erg* and *c-Myc* deletion (Fig. 5A) were compared to genomic loci bound by ERG and c-MYC to define target genes of the transcriptional network directly co-regulated by c-MYC and ERG (table S3). GO analysis was then undertaken to define the biological functions of these genes. This identified that the network was significantly enriched for genes involved in ribosome biogenesis as well as for genes involved in several metabolic processes (Fig. 5D). These findings were congruent with the GO analysis of up-regulated genes in human and murine *BCR::ABL1* B-ALL (Fig. 1F), thereby establishing the key roles for c-MYC and ERG in regulating these leukemia-associated changes. These data also identified direct regulation by ERG and c-MYC of genes involved in ribosome biogenesis such as the nucleolar proteins nucleophosmin (*Npm1*) (*32*) and fibrillarin (*Fbl*) (*33*) and subunits of the POL I complex (*Polr1b* and *Polr1c*) responsible for transcribing rRNA genes within the nucleoli (*34*) (Fig. 5E and table S3). This was in contrast to examples of genes specifically regulated by ERG or c-MYC (fig. S4B).

**Fig. 6.**
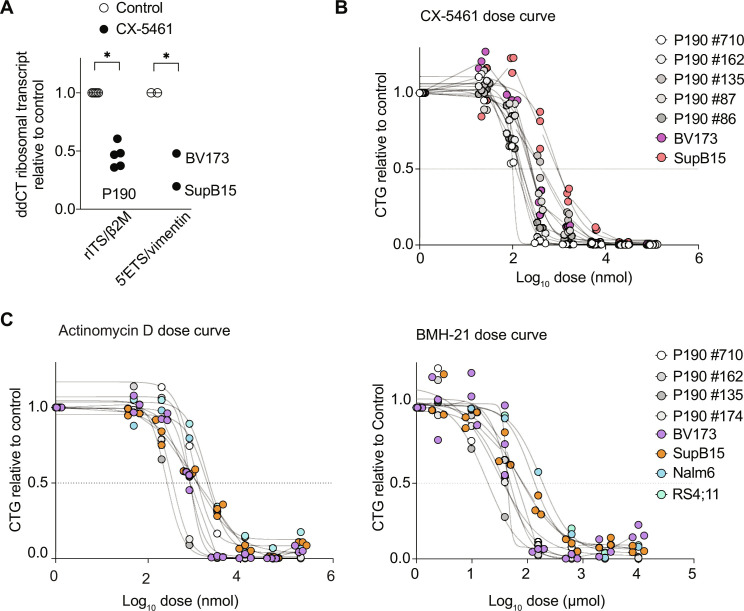
POL I inhibition in *BCR::ABL1* B-ALL cell lines. (**A**) On-target Pol I transcriptional inhibition with CX-5461 treatment at median inhibitory concentration (IC_50_) for 2 hours by reverse transcription polymerase chain reaction (PCR) of murine (47*S*/45*S* pre-rRNA ribosomal RNA (rRNA) internal transcribed spacer (rITS), relative to β-2-microglobulin (β2M), left) and human (47S/45S pre-rRNA 5′ETS, relative to vimentin, right) target genes in five independent murine (P190) and two independent human (BV173, SupB15) *BCR::ABL1* B-ALL cell lines. Mean of two independent experiments per cell line is shown. (**B**) Cell titer glow assay of murine and human *BCR::ABL1* B-ALL cell lines showing inhibition by CX-5461 at the doses indicated after 48 hour of treatment. The mean of three technical replicates per cell line is shown (points). IC_50_ of cell lines: P190 #86 (135 nM), P190 #87 (257 nM), P190 #135 (406 nM), P190 #162 (135 nM), P190 #710 (99 nM), BV173 (230 nM), and SupB15 (861 nM). (**C**) Cell titer glow assay of murine and human *BCR::ABL1* B-ALL cell lines showing inhibition by actinomycin D (left) and BMH-21 (right) at the doses indicated after 48 hours of treatment. The mean of three technical replicates per cell line is shown (points). IC_50_ of cell lines actinomycin D: P190 #710 (0.96 nM), P190 #162 (0.84 nM), P190 #135 (0.27 nM), P190 #174 (0.34 nM), BV173 (0.80 nM), SupB15 (1.05 nM), Nalm6 (1.92 nM), and RS4;11 (1.78 nM). IC_50_ of cell lines BMH-21: P190 #710 (29 nM), P190 #162 (46 nM), P190 #135 (22 nM), P190 #174 (40 nM), BV173 (43 nM), SupB15 (69 nM), Nalm6 (176 nM), and RS4;11 (120 nM).

As molecular validation of this analysis, we targeted POL I to disrupt ribosome biogenesis in *BCR::ABL1* B-ALL using the first-in-class small-molecule inhibitor of POL I transcription, CX-5461 (Pidnarulex). POL I transcription occurs in the nucleoli and produces the precursor rRNA (pre-rRNA) containing the sequences of the 18*S*, 5.8*S*, and 28*S* mature rRNA components of the ribosome (*35*, *36*). We confirmed on-target CX-5461–mediated inhibition of POL I transcription by measuring 47*S*/45*S* pre-rRNA levels in *BCR::ABL1* B-ALL cell lines (Fig. 6A). As inhibition of POL I transcription results in nucleolar disruption and the induction of nucleolar stress response, we assessed the effect of CX-5461 on nucleolar integrity using immunofluorescence staining for the nucleolar protein fibrillarin (FBL), a small nucleolar ribonucleoprotein that directs the methylation and processing of pre-rRNAs (*37*–*39*). In murine and human *BCR::ABL1* B-ALL cell lines, CX-5461 treatment resulted in reduced FBL staining that was localized in smaller punctate nucleolar domains as well as diffusely in the nucleoplasm, indicating significant nucleolar disruption (fig. S5, A and B). Consistent with the established action of CX-5461 in activating the nucleolar stress response (*39*, *40*), CX-5461 treatment was associated with up-regulation of the tumor suppressor TP53 (fig. S5C). CX-5461 treatment of murine and human *BCR::ABL1* leukemia cells caused dose-dependent growth inhibition at nanomolar drug concentrations (Fig. 6B), which has previously been shown to be due to induction of apoptosis in TP53 replete leukemia cells (*37*). Sensitivity to inhibition of POL I transcription was confirmed with two other POL I inhibitors, actinomycin D (*41*) and BMH-21 (*42*) (Fig. 6C). CX-5461 also demonstrated activity across other B-ALL genomic subtypes with dose-dependent growth inhibition of the *DUX4::IGH* (Nalm6) and *KMT2A::AF4* (RS4:11) B-ALL cell lines (fig. S5D), an observation that could also be extended with other POL I inhibitors (Fig. 6C).

## DISCUSSION

Ph^+^B-ALL remains a poor-prognosis genetic subtype of leukemia. The defining *t*(9;22) translocation generates the oncogenic BCR::ABL1 fusion protein that is the molecular driver of this leukemia and the primary target for small-molecule kinase inhibitors. However, despite the advent of TKI therapy combined with conventional intensive chemotherapy (*4*), leukemia relapse often occurs from preexisting clones carrying mutations in the tyrosine-kinase domain of the *BCR::ABL1* fusion gene (*43*) that render targeted kinase inhibition ineffective. There is a need to develop more effective therapeutic strategies to treat this disease, including identification of molecular targets other than BCR::ABL1 that can bypass resistance to TKI inhibitors and other potential mechanisms of chemotherapy resistance.

Transcriptional profiling of human and murine *BCR::ABL1* B-ALL identified members of two sequence-specific transcription factor families whose expression was high in Ph^+^B-ALL: *ERG*, a member of the ETS family, and the β helix–loop–helix family member *c-MYC* and its heterologous binding partner *MAX* (*44*). Notably, both ERG and c-MYC have also been shown to have critical roles in B cell development (*23*, *24*). Our data demonstrate that ERG and c-MYC are required in *BCR::ABL1* B-ALL initiation and leukemia cell expansion: Genetic deletion of *Erg* or *c-Myc* in the murine P190 transgenic model prevented or delayed disease onset. Moreover, in murine and human *BCR::ABL1* B-ALL cell lines, reduction of ERG or c-MYC levels constrained leukemia cell expansion and delayed leukemia development in transplanted mice. Examination of publicly available gene expression data suggests that ERG and c-MYC are co-expressed in other B-ALL subtypes in addition to *BCR::ABL1* B-ALL. Our observation that genetic reduction of *ERG* or *c-MYC* resulted in proliferative disadvantage in two other pre-B-ALL genomic subtypes provides preliminary evidence that our observations may extend more broadly to other forms of B-ALL, an observation that was also noted with variable gene dependency for *ERG* and c-*MYC* in B-ALL cell lines of different genomic subtypes in the Cancer Dependency Map initiative (*27*).

Genome-wide analysis of ERG- and c-MYC–dependent transcriptional changes in human and mouse *BCR::ABL1* B-ALL cell lines, combined with analysis of ChIP-seq datasets, defined a cooperative ERG- and c-MYC–dependent transcriptional network that included direct binding and transcriptional regulation of genes involved in metabolic pathways and ribosome biogenesis. Enrichment of genes involved in ribosome biogenesis, including components of the POL 1 transcription complex, allowed a molecular validation of the network via inhibition of POL 1 transcription using CX-5461, actinomycin D, and BHM-21, each of which caused dose-dependent growth inhibition of *BCR::ABL1* B-ALL cell lines at nanomolar drug concentrations. CX-5461 has previously been shown to selectively induce apoptosis in c-MYC driven leukemia cells (*37*). While an oncogenic role for c-MYC has been proposed via transcriptional amplification (*45*, *46*), prior evidence for c-MYC as a master regulator of ribosome biogenesis (*47*, *48*) and the observation that c-MYC–driven cancers are associated with hyperactivated POL I transcription (*35*, *36*) are particularly supported by our data, which now extends a similar role for c-MYC to *BCR::ABL1* B-ALL. While, in hematological disease, ERG has established oncogenic roles in myeloid malignancy (*49*–*53*) and T cell ALL (*54*, *55*), our finding of its cooperative role with c-MYC was unexpected and provides an important insight into the contribution of these two transcription factors in the biology of *BCR::ABL1* B-ALL. The transcriptional regulation by ERG and c-MYC of genes involved with metabolic pathways and ribosome biogenesis (*56*) may have broader implications for other malignancies, in which ERG is deregulated.

In summary, we have established essential roles for c-MYC and ERG in *BCR::ABL1*-driven B-ALL and defined an ERG- and c-MYC–dependent transcriptional network involved in regulation of metabolic processes and ribosome biogenesis in this disease. Together, our results validate an approach for defining essential transcriptional regulatory networks to elucidate important biological pathways in oncogenesis, among which previously unidentified vulnerabilities and therapeutic targets may emerge.

## MATERIALS AND METHODS

### Mice

Unless otherwise described, mice were generated on a C57BL/6 background. Mice with a conditional *Erg* knockout allele (*Erg^fl^*) were generated as previously described (*23*). Mice carrying a conditional c-*Myc* knockout allele (*24*) (*Myc^fl^*) were obtained from the Jackson Laboratory (*Myc^tm2Fwa^*). These mice were interbred with either *Rag1Cre* mice (*28*), in which Cre recombinase is expressed during lymphopoiesis from the CLP stage (*57*) or *CreERT2* mice (*29*), in which the expression of the Cre recombinase can be initiated by TAM treatment to generate *Rag1Cre^T/+^*;*Erg^fl/fl^*, *Rag1Cre^T/+^;Myc^fl/fl^*, *CreERT2^T/+^;Erg^fl/fl^, CreERT2^T/+^;Myc^fl/fl^*, and wild-type control littermates. Subsequently these were crossed to P190 transgenic mice (*13*). Mice were co-housed in a barrier facility and analyzed from 6 to 18 weeks of age. Male and female mice were used. The primers used for genotyping are provided in table S4. NOD.Cg-*Prkdc^scid^IL2rg^tm1Wjl^*/Szj (NSG) female mice were obtained from the Jackson Laboratory and co-housed in individually ventilated cages in a specific pathogen–free facility. Experimental procedures were approved by The Walter and Eliza Hall Institute of Medical Research Animal Ethics Committee.

### Cell culture and viral transduction

Human BV173 cells were maintained in RPMI 1640 supplemented with 10% fetal calf serum (FCS; Gibco, Invitrogen). SupB15 cells were cultured in Iscove's modified Dulbecco's medium (IMDM) supplemented with 20% FCS. Murine P190 leukemia cell lines were generated as described previously (*23*) and maintained in IMDM supplemented with 10% FCS, 50 μM β-mercaptoethanol, and murine interleukin-7 (10 ng/ml). Lentiviral transduction of human cell lines with VSVg-pseudotyped viruses to enable expression of Cas9- or gene-specific single-guide RNAs (table S4) was performed as described (*58*).

### In vitro cell assays

To measure adenosine 5′-triphosphate levels after 4-OHT, CX5461, actinomycin D, or BMH-21 treatment, 2500 cells were seeded per well in triplicates in the indicated drug concentrations and analyzed after 48 to 96 hours of culture as indicated by the CellTiter-Glo Luminescence Assay (Promega). For in vitro competition cultures, cells transduced with a Dox-inducible fluorescently labeled short hairpin RNA/guide RNA vector were co-cultured with uninfected parental cells, split into two aliquots and treated with Dox (100 ng/ml) or dimethyl sulfoxide (DMSO). The relative frequency of transduced and non-transduced cells was measured over time by flow cytometry.

### Flow cytometry

For flow cytometric analysis, single-cell suspensions were prepared in balanced salt solution (0.15 M NaCl, 4 mM KCl, 2 mM CaCl_2_, 1 mM MgSO_4_, 1 mM KH_2_PO_4_, 0.8 mM K_2_HPO_4_, and 15 nM Hepes supplemented with 2% bovine calf serum). Cells were washed, stained with fluorophore-conjugated antibodies (see table S5), and analyzed or sorted on a BD LSRFortessa or BD FACSAria III, respectively. Dead cells were excluded by staining with FluoroGold (AAT Bioquest), and data analysis was performed using FlowJo 10.4 (Becton Dickinson). To determine total cell numbers, an aliquot of the single-cell suspension was mixed with a defined number of allophycocyanin (APC^+^) beads. The ratio of cells/bead was used to determine the total cell count per femur.

### B-ALL transplantation studies

For B-ALL transplantation studies, cell lines derived from leukemic *P190^T/+^;CreER*^T/+^;*Erg^fl/fl^* and *P190^T/+^;CreER^T/+^;c-Myc^fl/fl^* mice were transplanted into sublethally irradiated (4.5gray) C57BL/6-Ly.1/Ly5.2 mice by intravenous injection of 1 × 10^6^ cells. R26-CreERT2 (CRE-ER) activity was either induced in vitro by addition of 4-OHT for 5 days before transplantation (pre4-OHT; 100 nM 4-OHT) or in vivo by administering TAM via oral gavage to mice on day 3 to 6 after transplantation (TAM; 0.6 mg/g body weight/day). For xenograft assays, 1 × 10^6^ Cas9^+^ sguide^+^ BV173 cells were intravenously transplanted into NOD-Scid IL2r/J mice. Sguide expression was induced in vivo at day 7 by administration of Dox via standard food pellets (Specialty Feeds, 600 mg/kg). Pretreated cells had, in addition, been treated with Dox for 5 days in vitro before transplantation (PreDox; 100 ng/ml).

### Immunoblots and co-immunoprecipitation

For Western blotting, cells were lysed in KALB lysis buffer [1% Triton X-100, 150 mM NaCl, 50 mM tris-HCl (pH 7.4), 1 mM EDTA, 1 mM phenylmethylsulfonyl fluoride, 2 mM Na_3_VO_4_, 10 mM NaF, and complete protease inhibitors (Roche)]. Proteins were separated in 4 to 12% Bis-Tris NuPAGE protein gels (Invitrogen) under reducing conditions, transferred onto a Immobilon-P membrane (Millipore), and immunoblotted with primary antibodies (see table S5), followed by incubation with secondary horseradish peroxidase–conjugated antibodies and visualization by enhanced chemiluminescence. For co-immunoprecipitations, cells were lysed as described above. Cell lysates were incubated with 0.25 μg of specific antibody or IgG control for 2 hours, Protein A slurry was added, and the mix was incubated for >3 hours before Protein A beads were washed three times in KALB lysis buffer and protein was eluted and then analyzed by Western blot.

### Real-time PCR analysis

rRNA transcription by POL I in mouse and human *BCR::ABL1* leukemia cells was assessed at the *47S*/*45S rRNA ITS* and *47S/45S rRNA 5′ETS* genes relative to β*2m* and *VIM* genes, respectively (see table S4). RNA was reverse-transcribed to cDNA using random hexamer primers (Promega, Madison, USA) and Superscript III reverse transcriptase (Invitrogen) according to the manufacturer’s instructions. Quantitative polymerase chain reaction (PCR) was performed using SYBR green reagents (Applied Biosystems, USA) on the ViiA 7 real-time PCR system (Thermo Fisher Scientific, USA), and relative expression of rRNA genes after treatment with CX-5461 compared to housekeeping genes was expressed relative to DMSO-treated cells using the 2^−ΔΔCT^ method (*59*).

### RNA sequencing

Total RNA was extracted using the RNeasy Plus minikit (Qiagen) from murine *BCR::ABL1* B-ALL cell lines and B220^+^ selected pre-B cells. RNA (500 ng) was used to generate cDNA libraries using TrueSeq Stranded mRNA kits (Illumina). Sequencing was performed on a Hi-Seq2500 or NovaSeq sequencing system (Illumina) to generate 100 bp single-end reads. Pseudo-biological replicates were sequenced for each *BCR::ABL1* B-ALL cell line. Reads were aligned to the GRCm38/mm10 build of the *Mus musculus* genome using Rsubread align function, and read counts were summarized at the gene level (*60*). Genes were filtered as non-expressed if they were assigned 0.5 counts per million mapped reads (CPM) in fewer than two libraries. Counts were transformed to log_2_-CPM and the mean-variance relationship estimated using the voom function in limma (*61*). Library sizes were trimmed mean on M-values (TMM)–normalized, and differential expression was assessed using quasi-likelihood *F* tests (*62*). Genes were called differentially expressed if they achieved a false discovery rate of 0.05. For plotting purposes, counts were converted to reads per kilobase of transcript per million mapped reads (RPKM) using the rpkm function in limma. These data have been deposited in Gene Expression Omnibus database (accession number GSE213791). For clonotype analysis, fastq files from pre-B cells from individual mice or pooled from *n* = 3 mice for samples taken at 5 weeks and primary leukemia were analyzed using the MiXCR software package (3.0.6) (*63*). The frequency of the 10 most prevalent clonotypes was normalized to frequency per mouse.

### ChIP-seq analysis

Publicly available FASTQ files for ERG (GSM3895108), c-MYC (GSM1234475), H3K4me3 (GSM2255547), and H3K27Ac (GSM2255552) ChIP-seq experiments were aligned to the mm10 mouse reference genome (GRCm38, December 2011) using Rsubread (*64*). Peak calling was performed using MACS2 (*65*) against input FASTQ files.

### Gene network analysis

All ERG and c-MYC ChIP-seq peaks mapping to differentially expressed genes in both *P190^T/+^;CreERT2^T/+^;Erg*^Δ*/Δ*^ and *P190^T/+^;CreERT2^T/+^;c-Myc^Δ/Δ^* cell lines within 10 kb of the TSS were identified. Peaks inside the gene body were annotated as “proximal targets,” peaks overlapping the TSS were labeled as promoter regulated targets, peaks less than 3 kb upstream or downstream of the TSS were labeled as putative promoter regulated targets, and peaks more than 3 kb upstream or downstream TSS were labeled as putative distal targets (see table S3). GO annotation of differentially expressed genes was performed and underwent expert manual curation. The network was constructed using igraph CRAN package (*66*) and exported to Cytoscape (*67*) for customization using RCy3 (*68*) R/Bioconductor package.

### Visualization of RNA-seq, ChIP-seq, and ATAC-seq data

RNA-seq and ChIP-seq files were converted to BigWig files using deepTools (version 2) (*69*) and uploaded to Cyverse (www.cyverse.org) for visualization in UCSC Genome Browser (*70*) (genome.ucsc.edu) or Integrative Genomics Viewer (*71*).

### Immunofluorescence analysis

Suspension cells were fixed in a 4% paraformaldehyde solution for 10 min and then cytospun onto pre-charged Super Frost Plus Slides (Menzel Gläser) using a double cytology funnel. Slides were permeabilized for 10 min in ice-cold phosphate-buffered saline (PBS; 0.1 M, pH 7.4) containing 0.3% Triton X-100. Following permeabilization, cells were washed three times with PBS (5 min each with gentle rocking on a lab shaker) and blocked in PBS consisting of 5% goat serum and 0.3% Triton X-100 for 30 min at room temperature. Next, slides were incubated with primary antibodies (see table S5) in 1% bovine serum albumin (BSA)/PBS for 1 hour at 37°C in a humidified chamber. Following staining, slides were washed three times with PBS (5 min each with gentle rocking) and then subsequently incubated with secondary antibodies (see table S5). Secondary antibodies were diluted 1:600 in 1% BSA/PBS, and slides were stained for 1 hour at 37°C. Last, cells were washed in PBS and counterstained for 10 min with 4,6-diamidino-2-phenylindole (1 μg/ml; Sigma-Aldrich) before being mounted with glass coverslips using VECTASHIELD Anti-fade Mounting Medium (Vector Laboratories).

Fluorescent confocal images were acquired using a Nikon C2 laser scanning confocal microscope system (Nikon, Melville, NY) equipped with a 60× oil immersion objective and NIS-Elements software (Nikon, Melville, NY) for acquisition of the images. A maximal intensity projection of a *Z*-stack was than generated using the software program ImageJ (1.47v, National Institutes of Health). Images were analyzed using CellProfiler version 3.1.9 (Broad Institute) using the same manually set parameters and thresholds. For statistical analysis, mean signal intensity data values were normalized to the median of each respective vehicle control and the data plotted using GraphPad Prism software (version 7) performing a two-sided unpaired Mann-Whitney test where appropriate.

### Statistical analysis

Statistical significance was analyzed using one-way analysis of variance (ANOVA) with Dunnett’s correction for multiple comparison, *t* test, and Gehan-Breslow-Wilcoxon test or as indicated in the figure legends (GraphPad Prism software version 8). *P < 0.05; **P < 0.005; ***P < 0.001; ****P < 0.0001. Data are presented as means ± SD.
