# Supplementary material for: ERG and c-MYC regulate a critical gene network in BCR::ABL1-driven B cell acute lymphoblastic leukemia
Source: Sci Adv. 2024 Mar 8;10(10):eadj8803. doi: 10.1126/sciadv.adj8803 (PMC10923517; doi:10.1126/sciadv.adj8803)
Supplement: Supplementary file 1 — Figs. S1 to S5 Legends for tables S1 to S3 Tables S4 and S5 Legend for data S1 [file sciadv.adj8803_sm.pdf]

Science Advances
AAAS

Supplementary Materials for

ERG and c-MYC regulate a critical gene network in BCR::ABL1-driven B cell acute lymphoblastic leukemia

Kira Behrens *et al.*

Corresponding author: Ashley P. Ng, ang@wehi.edu.au

Sci. Adv. **10**, eadj8803 (2024)
DOI: 10.1126/sciadv.adj8803

The PDF file includes:

Figs. S1 to S5
Legends for tables S1 to S3
Tables S4 and S5
Legend for data S1

Other Supplementary Material for this manuscript includes the following:

Tables S1 to S3
Data S1

Fig. S1.

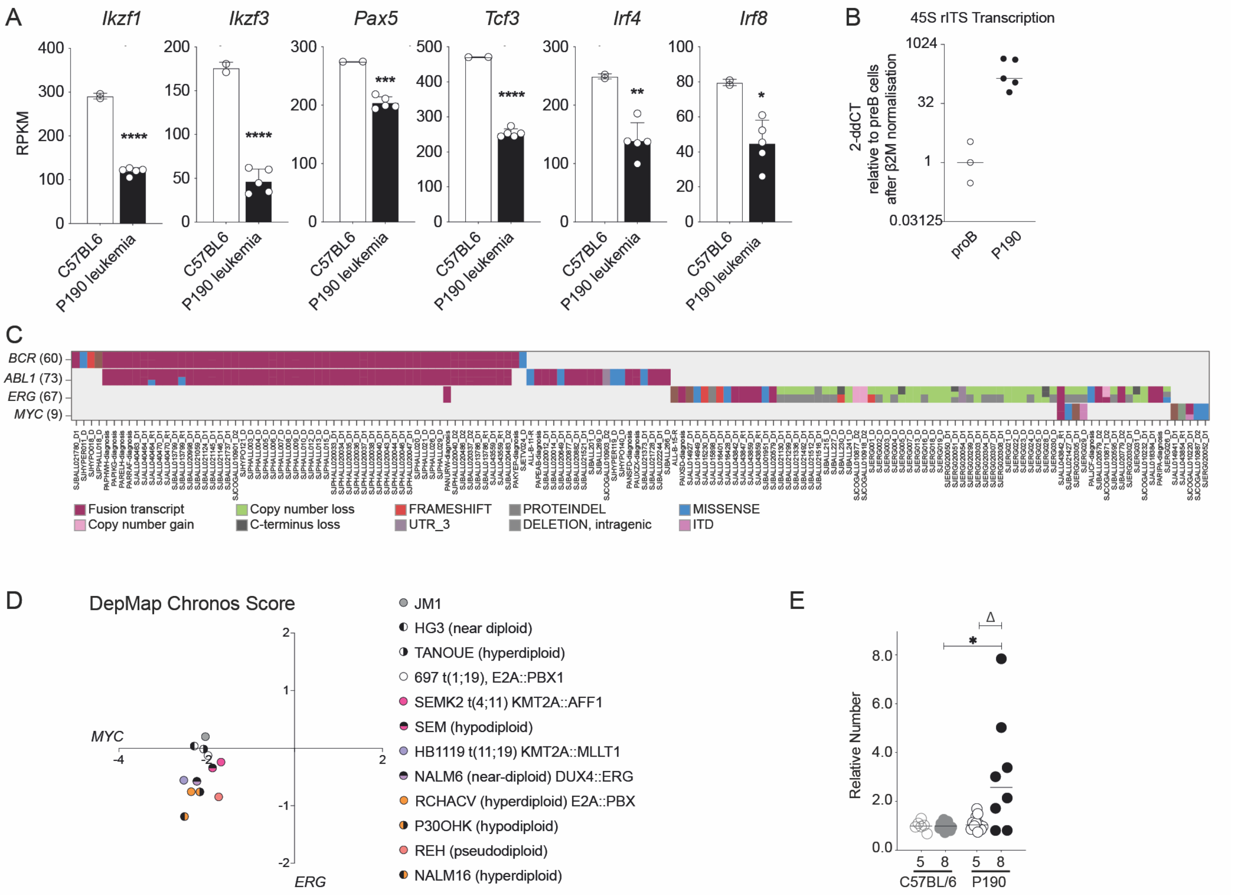

Transcriptional changes, genomic lesions and genetic dependencies in pre-B ALL. A) Gene expression changes for murine B-lineage transcription factors in P190 B-ALL (RPKM, reads per kilobase per million mapped reads from RNA-seq data). *P*-values were calculated by unpaired t-test. *, $P \leq 0.05$; **, $P \leq 0.01$; ***, $P \leq 0.001$; ****, $P \leq 0.0001$. **B**) 45S pre-rRNA rITS transcription relative to beta-2-microglobulin (β2M) in three independent proB cell samples and five independent murine P190 B-ALL cell lines. Mean of two independent experiments per cell line shown. $P < 0.036$ Mann-Whitney test. C) Genomic lesions in *BCR*, *ABL1*, *ERG* and *c-MYC* alleles in paediatric B-cell acute lymphoblastic leukaemia from the St Jude Paediatric Cancer Consortium *(73)*. Loss of function *ERG* and *c-MYC* alleles are not observed in *BCR*::*ABL1* or *ABL1* rearranged B-cell leukaemias. Copy number loss/intragenic deletion of *ERG* are associated with the *DUX4::IGH* genomic subtype of B-ALL. **D)** CRISPR-Cas9 gene dependency screening from the Cancer Dependency Map Initiative (https://depmap.org/portal/). Chronos Score for B-ALL cell lines for *ERG* and *MYC* dependency by CRISPR (Chronos score of -1 is equivalent to an essential gene). **E)** Relative number of pre-B cells in P190 mice relative to C57BL/6 wild-type control mice at 5 (n= 6-11) and 8 weeks (n= 8-14) of age (* $P_{adj} = 0.0004$; Δ $P_{adj} = 0.0015$ by Sidak's multi-comparison test).

Fig. S2.

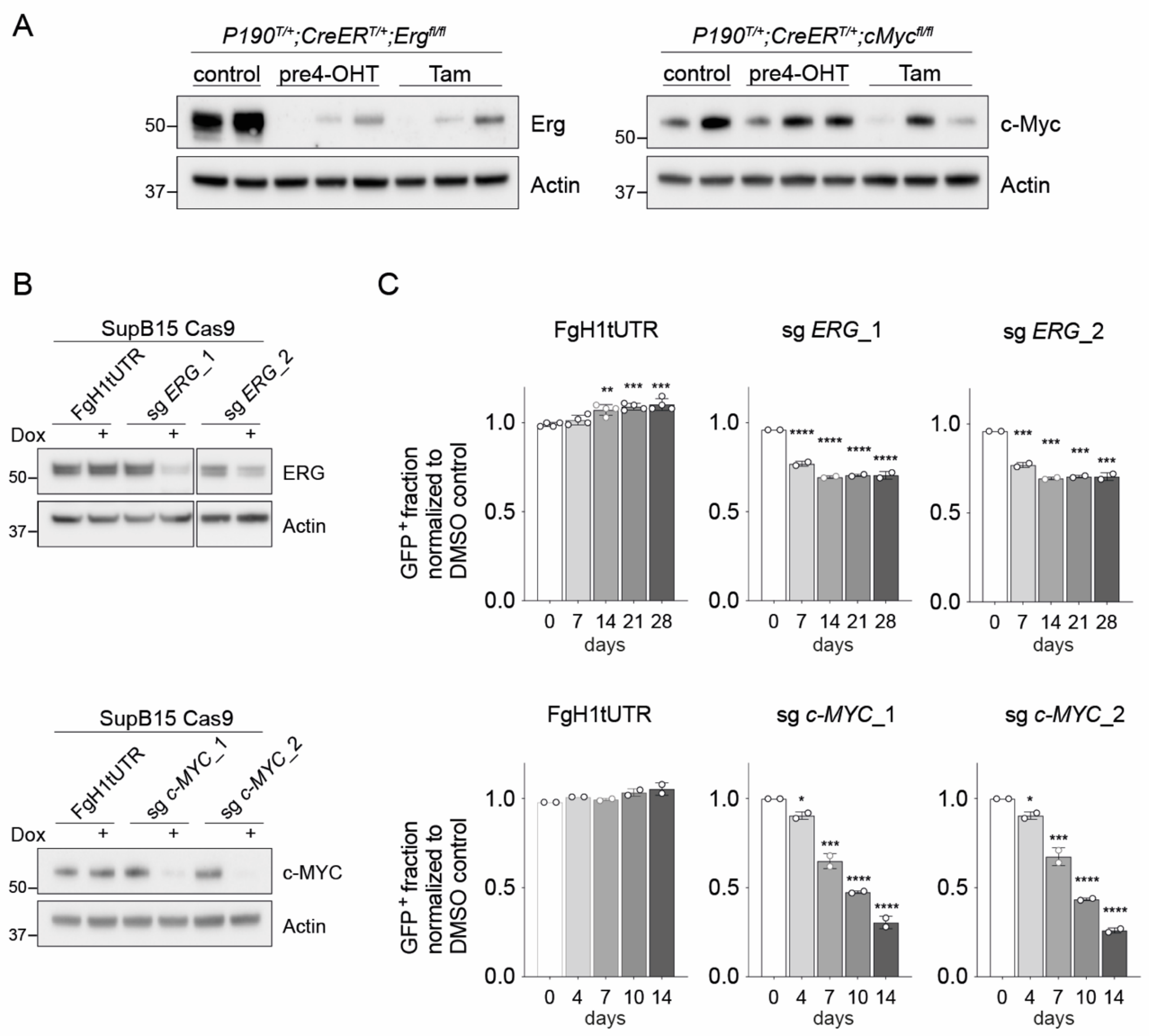

The functional role of ERG and c-MYC in the SupB15 *BCR::ABL1* cell line. A) Western blot analysis of the levels of Erg (left) or c-Myc (right) in whole bone marrow lysates of leukaemic mice (see Fig. 3E) transplanted with *P190*$^{T/+}$*; CreER*$^{T/+}$*; Erg*$^{fl/fl}$ or *P190*$^{T/+}$*; CreER*$^{T/+}$*; c-Myc*$^{fl/fl}$ cells and treated as indicated. **B**) Western blot analysis of ERG and c-MYC in SupB15 cells expressing two independent guide RNAs against *ERG* or c-*MYC*. **C**) *In-vitro* proliferation of Dox-treated SupB15 Cas9 sg *ERG*, SupB15 Cas9 sg c-*MYC* or SupB15 Cas9 control cells containing the empty FgH1UTR vector (https://www.addgene.org/70183/), relative to DMSO-treated control cells, measured on the days indicated following initiation of culture. Shown are the mean of two independent experiments. Error bars represent SD. *P*-values were calculated by one-way ANOVA with Dunnett's multiple comparison test. *, $P \leq 0.05$; **, $P \leq 0.01$; ***, $P \leq 0.001$; ****, $P \leq 0.0001$.

Fig. S3.

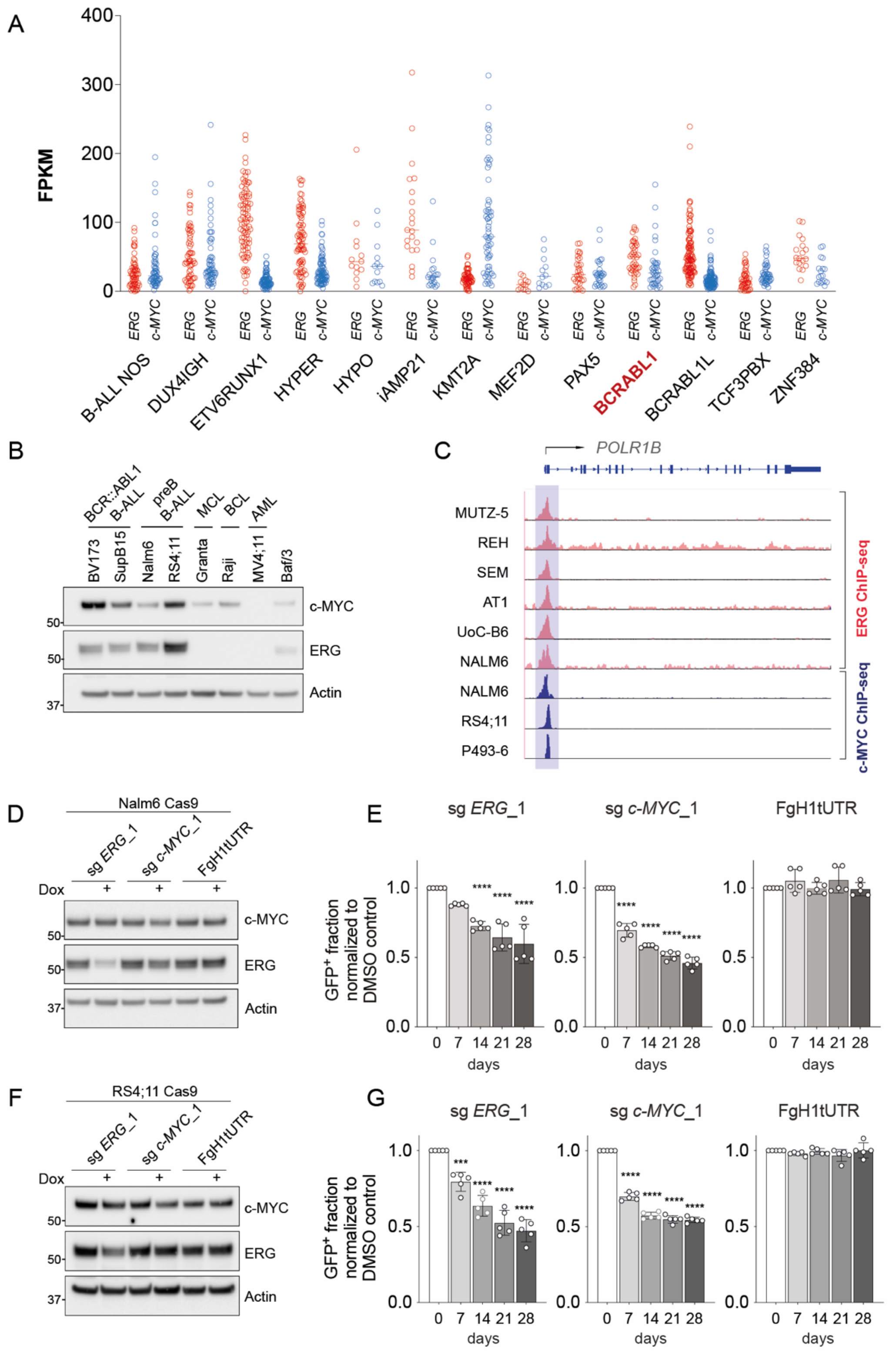

Expression and function of ERG and c-MYC in B-ALL genomic subtypes. A) Expression of *ERG* and c-*MYC* by RNAseq FPKM across B-ALL genomic subtypes (St Jude, PeCan, accessed 31st May 2023). B-ALL NOS, B-ALL not otherwise specified; DUX4IGH, B-ALL *DUX4::IGH*;

ETV6RUNX1, B-ALL *ETV6::RUNX*; HYPER, B-ALL Hyperdiploidy; HYPO, B-ALL Hypoploidy; iAMP, B-ALL iAMP21; KMT2A, B-ALL *KMT2A* rearrangement; MEF2D, B-ALL *MEF2D* rearrangement; PAX5, B-ALL *PAX5* alteration; BCRABL1, B-ALL *BCR::ABL1*; BCRALB1L, B-ALL *BCR::ABL1-like*, TCF3PBX, B-ALL *TCF3::PBX*; ZNF384, B-ALL *ZNF384* rearrangement. **B**) Western blot analysis of ERG or c-MYC in indicated cell lines: BCR::ABL1 B-ALL, preB-ALL, MCL, Mantle Cell Lymphoma, BCL, Burkitt Lymphoma, AML, acute myeloid leukaemia and Baf/3 control. **C)** ChIP-seq tracks of the *POLR1B* gene locus showing ERG and c-MYC binding to promoter regions in indicated cell lines: MUTZ-5 (t(12;13), GSM5663978), REH (*ETV6::RUNX1*, GSM5663979), SEM (*MLL::AF4*, GSM5663980), AT1 (*E2A::PBX1*, GSM6398674), UoC-B6 (*ETV6::RUNX1*, GSM5663981), Nalm6 (*DUX4::IGH*, GSM6398675), P493-6 (EREB2-5 EBV, cMYC-tet on, GSM894060). (**D,F)** Western blot analysis of ERG or c-MYC expression in Nalm6 (B-ALL, *DUX4::IGH*) (**D**) or RS4;11 (B-ALL, *KMT2A* rearranged) (**F**) cells transduced to express Cas9 and Dox-inducible guide RNAs targeting *ERG* (sg *ERG*_1), c-*MYC* (*sg c-MYC*_1) or an empty vector (FgH1UTR). Actin serves as loading control. Cells were treated with 100 ng/mL Dox (+) or vehicle for 96 hours as indicated. **E,G)** *In vitro* proliferation of Dox-treated *ERG* sguide, *MYC* sguide or empty vector expressing Nalm6 Cas9 (**E**) or RS4;11 Cas9 (**G**) cells relative to DMSO-treated control cells. Shown are the mean of three independent experiments. Error bars represent SD. *P*-values were calculated by one-way ANOVA with Dunnett's multiple comparison test. **, $P \leq 0.01$; ***, $P \leq 0.001$; ****, $P \leq 0.0001$.

Fig. S4.

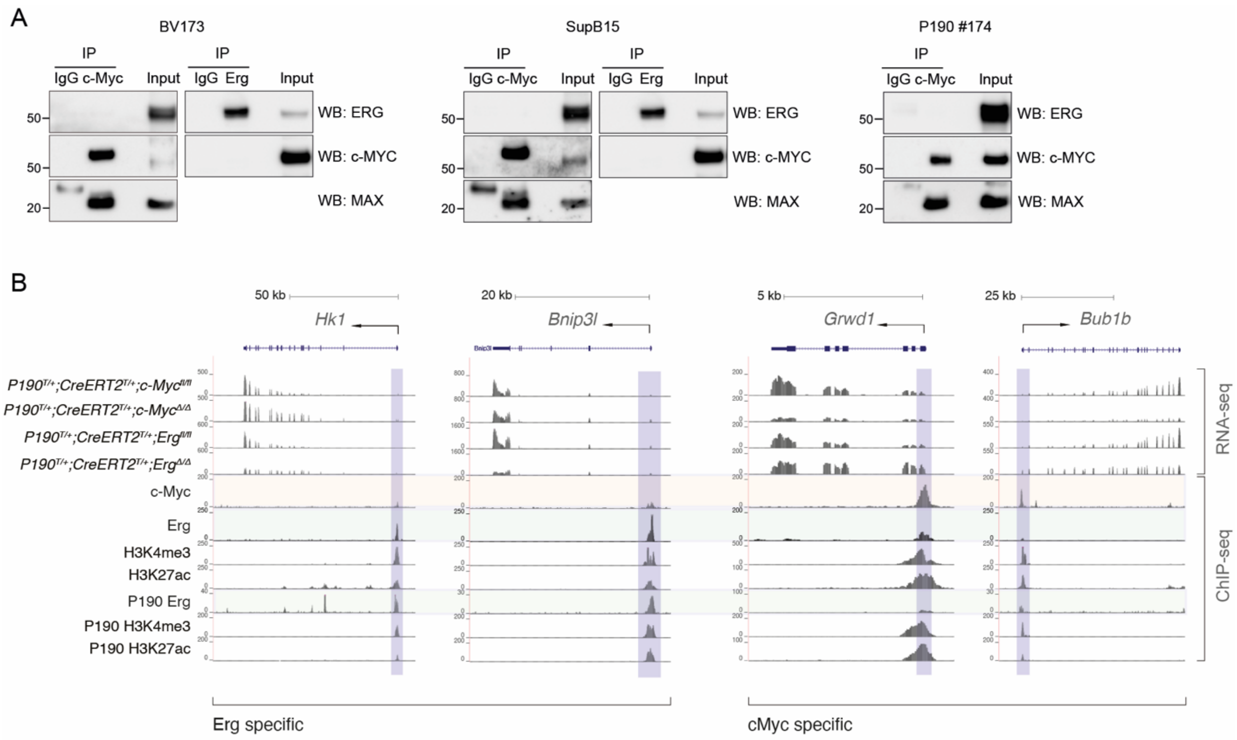

ERG and c-MYC independence by immunoprecipitation and at specific genomic loci in *BCR::ABL1* B-ALL. A) Immunoprecipitations of ERG or c-MYC in human (B173, SupB15) and murine B-ALL cell lines indicate no direct interaction between the two transcription factors. ERG or c-MYC were immunoprecipitated using specific antibodies and protein A Sepharose beads and analysed by Western Blot analysis. MAX serves as an internal control for c-MYC co-immunoprecipitation. **B**) RNA-seq and ChIP-seq tracks of *Hk1, Bnipl3, Grwd1 and Bub1b* gene loci showing transcriptional changes associated with Erg ($P190^{T/+};CreER^{T/+};Erg^{\Delta/\Delta}$) or c-Myc ($P190^{T/+};CreER^{T/+};Myc^{\Delta/\Delta}$) deletion compared to $P190^{T/+};CreER^{T/+};Erg^{fl/fl}$ *or* $P190^{T/+};CreER^{T/+};Myc^{fl/fl}$ controls, and ERG or c-MYC binding to promoter regions (highlighted blue) defined by H3K4me3 (GSM2255547) and H3K27Ac marks (GSM2255552), with tracks for independent ChIP-seq undertaken in the P190 *BCR::ABL1* cell line for ERG, H3K4me3 and H3K27Ac shown.

Fig. S5.

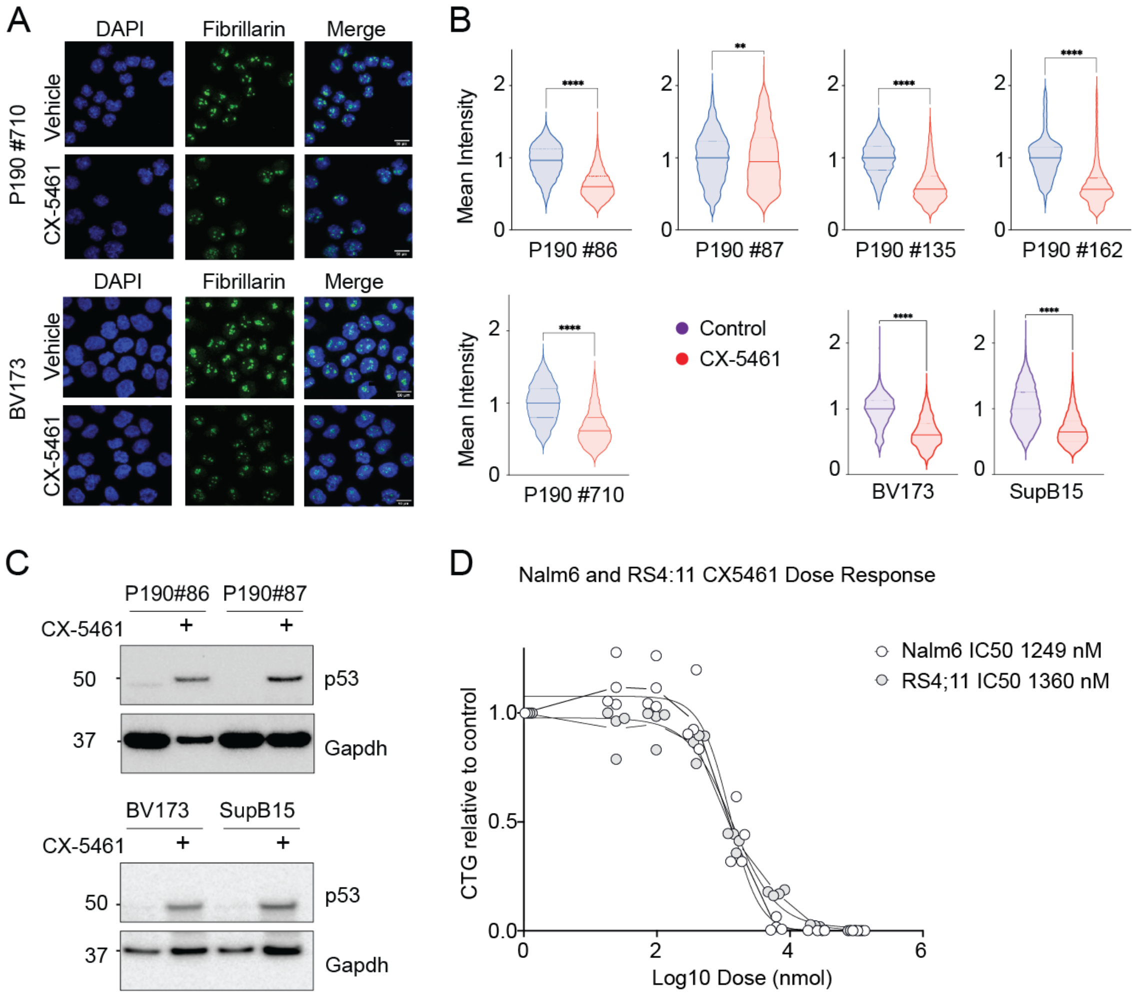

The effect of CX-5461 POL I inhibition in B-ALL. A) Representative immunofluorescence images of fibrillarin nucleolar protein in murine (top) and human (bottom) *BCR::ABL1* B-ALL cell lines treated with vehicle or CX-5461 at IC50 for 2 hours. Merged images were captured at 60x magnification with DAPI stained nuclei (blue) and Fibrillarin (green) (scale bar = 10µm). **B)** Quantitation of fibrillarin signal intensity in five independent murine (top) and two independent human *BCR::ABL1* B-ALL cell lines (bottom) was performed using CellProfiler (Version 4.2.1, Broad Institute) in which at least 200 cells per treatment condition were examined over three independent experiments. Mean signal intensity was normalized to the median value of corresponding vehicle control (****, $P \leq 0.0001$; ** $P \leq 0.01$, two-sided unpaired Mann–Whitney test). **C)** Induction of p53 in murine (top) and human (bottom) *BCR::ABL1* B-ALL cell lines treated with CX-5461 at IC50 plus QVD-OPH (5nM) (caspase inhibitor to prevent apoptosis) for 48 h. **D)** Cell titre glow assay of Nalm6 and RS4;11 cell lines showing growth inhibition by CX-5461 at the doses indicated after 48 h of treatment. The mean of three replicates per cell line is shown (points). IC50 of cell lines: Nalm6 (1249 nM), RS4;11 (1360 nM).

Table S1: Differential expression between P190 pre-B cell leukaemia and wild-type pre-B cells and GO Term analysis. (Excel File)

Table S2: RNA-seq differential expression in *P190*$^{T/+}$*;CreERT2*$^{T/+}$*;Erg*$^{\Delta/\Delta}$ and *P190*$^{T/+}$*;CreERT2*$^{T/+}$*;Myc*$^{\Delta/\Delta}$ cell lines. (Excel File)

Table S3: ERG and c-MYC gene regulatory network in *BCR::ABL1* B-ALL and classification of genes involved in ribosome biogenesis. (Excel File)

Table S4.
Genotyping and RT-PCR primers and sGuide sequences.

**Target**	**Direction**	**Sequence**
**primers**		
**genotyping**		
P190	Fwd	AGAGATCAAACACCCTAACCT
P190	Rev	CCAAAGCCATACTCCAAATGC
Myc	Fwd	GCTAGGAAGACTGCGGTGAG
Myc	Rev	GCCACTGCACCAGAGACC
**RT-PCR**		
47S/45S pre-rDNA ITS	Fwd	CCGGCTTGCCCGATTT
47S/45S pre-rDNA ITS	Rev	GCCAGCAGGAACGAAACG
47S/45S pre-rRNA 5'ETS	Fwd	GGCGGTTTGAGTGAGACGAGA
47S/45S pre-rRNA 5'ETS	Rev	ACGTGCGCTCACCGAGAGCAG
B2m	Fwd	TTCACCCCCACTGAGACTGAT
B2m	Rev	GTCTTGGGCTCGGCCATA
Vimentin	Fwd	AGAGAACTTTGCCGTTGAAGCT
Vimentin	Rev	GAAGGTGACGAGCCATTTCC
**sguides**		
sg *ERG_1*		TCCCCAAGATGACGGATCCCGACG
sg *ERG_2*		TCCCGACACCGTTGGGATGAACTA
sg *c-MYC_1*		TCCCAGAGTGCATCGACCCCTCGG
sg *c-MYC_2*		TCCCGCTGCACCGAGTCGTAGTCG

Table S5.

Antibodies used for flow cytometric analysis, immunofluorescence, immunoprecipitation, and Western blot analysis.

**Antigen**	**Conjugate**	**Clone**	**Supplier**
**Western Blots**			
Erg		9FY	Biocare Medical
Erg		EPR3864	Abcam
Erg		EPR3864(2)	Abcam
Max		EPR19352	Abcam
Myc		Y69	Abcam
Myc		E5Q6W	Cell Signaling Technology
Actin	HRP	C-4	Santa Cruz Biotechnology
Actin	HRP	I-19	Santa Cruz Biotechnology
Trp53		CM5	Leica Biosystems
Gapdh	HRP	EPR16891	Abcam
Donkey anti-Rabbit IgG	HRP		GE Healthcare
Goat anti-Mouse Ig	HRP		Southern Biotech
Mouse anti rabbit IgG (light chain specific)	HRP	D4W3E	Cell Signaling Technology
Mouse anti-Rabbit IgG (conformation specific)	HRP	L27A9	Cell Signaling Technology
**Immunofluorescence**			
Fibrillarin		polyclonal	Abcam
Goat anti-Rabbit IgG	A488		Life technologies
**Flow Cytometry**			
B220	APC, APC-Cy7	RA3-6B2	BD Pharmingen
B220	BV650	RA3-6B2	BD Horizon
CD19	A700	1D3	WEHI Antibody Facility
CD19	PE-Cy7	1D3	BD Pharmingen
CD24	PerCP-Cy5.5	M1/69	BD Pharmingen
CD43	PE-Cy7	S7	BD Pharmingen
IgD	PE	11-26c.2a	BD Pharmingen
IgM	APC, FITC	5.1	WEHI Antibody Facility
human CD45	APC	HI30	Invitrogen
murine CD45.1	PE-Cy7	A20	BD Pharmingen
murine CD45.2	A647	S450	WEHI Antibody Facility

A488, AlexaFluor488; A647, AlexaFluor647; A700, AlexaFluor700; BV650, Brilliant Violet-650; FITC, fluorescein isothiocyanate; APC, Allophycocyanin; PE, phycoerythrin; PerCP-Cy5.5, peridinin chlorophyll protein-cyanine5.5 tandem, PE-Cy7, phycoerythrin-cyanine7 tandem, APC-Cy7, Allophycocyanin-cyanine7 tandem

Data S1. Raw data points used to generate figures in this manuscript.
